# Benchmark Datasets for Bilateral Lower-Limb Neuromechanical Signals from Wearable Sensors during Unassisted Locomotion in Able-Bodied Individuals

**DOI:** 10.3389/frobt.2018.00014

**Published:** 2018-02-19

**Authors:** Blair Hu, Elliott Rouse, Levi Hargrove

**Affiliations:** ^1^Center for Bionic Medicine, Shirley Ryan AbilityLab, Chicago, IL, United States; ^2^Department of Biomedical Engineering, Northwestern University, Evanston, IL, United States; ^3^Department of Mechanical Engineering, University of Michigan, Ann Arbor, MI, United States; ^4^Department of Physical Medicine and Rehabilitation, Northwestern University, Chicago, IL, United States

**Keywords:** gait, locomotion, biomechanics, electromyography, benchmark

## Introduction

The field of assistive robotics has experienced rapid growth in the number of and capabilities of wearable lower limb assistive devices, which include robotic exoskeletons, orthoses, and prostheses. These devices have shown promising potential to restore motor function to individuals with gait impairments by providing locomotion assistance. Although many devices have already demonstrated impressive performance in a variety of real-world conditions, comparing their performance objectively and improving their controllability remain challenging for several reasons. First, the outcome measures (e.g., joint kinematics, metabolic cost, clinical scores, and prediction accuracy) used by studies demonstrating improved walking ability with an assistive device are not consistent. Second, many studies only use treadmill walking or do not collect data from a variety of locomotor activities due to constraints in a device’s mechatronic design and/or control system. Third, many devices are in the process of commercialization, so testing data are seldom shared with the research community. In addition, many devices implement their own unique control frameworks that are not generic enough to conveniently implement on other hardware. Therefore, we expect improving access to device-agnostic neuromechanical signals during walking-related activities (from which researchers could develop and test novel control strategies before implementation on hardware) will be valuable to the field of wearable lower limb assistive devices.

Meanwhile, many benchmarks for the biomechanics of able-bodied human locomotion without an assistive device have already been established, some of which are publicly available. The gold standard for high-resolution biomechanical gait analysis is marker-based optical motion capture with ground reaction force measurement. Decades ago, seminal work from Winter (1983) used these techniques to introduce an inter-subject biomechanical analysis of level ground walking (LW) at different speeds. Their normative gait dataset includes electromyography (EMG) and joint kinematic and kinetic patterns and has since been expanded by other researchers to include more subjects and strides (e.g., Kadaba et al., 1990; Kirtley, 2014). The steady-state biomechanics of other common locomotor activities such as ascending and descending stairs and sloped surfaces of different geometries have also been reported in separate studies using similar techniques but these data are not as accessible to researchers (e.g., McFadyen and Winter, 1988; Riener et al., 2002; Lay et al., 2006, 2007; Protopapadaki et al., 2007; Franz et al., 2012).

Human locomotion is most accurately quantified by joint kinematics, kinetics, and EMG using traditional laboratory-based instrumentation and techniques developed for biomechanical gait analysis. However, the exciting potential of wearable lower limb robotics lies in its promise to bring these devices closer to everyday life, where alternative techniques are required to more ubiquitously measure neuromechanical signals during walking-related activities. Methods to more freely measure human movement have been developed in the field of human activity recognition (HAR), which aims to use continuous streams of sensor data to recognize and monitor common activities of daily living such as sleeping, walking, exercising, and manipulating objects. As a result, HAR has produced an abundance of publicly available datasets. These repositories are valuable because they contain many different types of activity information from many subjects; however, they are not very suitable for more systematic characterization of normal locomotion. Sometimes, HAR datasets are collected from impaired populations or during more natural, but complex combined movements for which the ground truth activity is more ambiguous. Some datasets are collected using minimal instrumentation (e.g., smartphone only), which is convenient but incomplete. By contrast, others rely on non-portable instrumentation (e.g., optical motion capture or video), which is highly accurate but not representative of biomechanical signals accessible for controlling a device in a more ecological setting. Also, many only contain single modalities (e.g., kinematics but no EMG) and/or use lower sampling rates that may be insufficient for certain online control schemes.

To the best of our knowledge, there still does not exist a publicly available database of kinematic and EMG data simultaneously recorded from wearable sensors as able-bodied individuals freely transition between several distinct locomotor activities. To address some of these aforementioned limitations and provide relevant reference data for researchers in the field of wearable lower limb assistive devices, we introduce a device-agnostic benchmark dataset of bilateral neuromechanical signals called ENcyclopedia of Able-bodied Bilateral Lower Limb Locomotor Signals (ENABL3S). The dataset contains bilateral EMG and joint and limb kinematics recorded from wearable sensors for 10 able-bodied individuals as they freely transitioned between sitting, standing, and several walking-related activities [level ground, stair ascent (SA)/stair descent (SD), and ramp ascent (RA)/ramp descent (RD)]. Although these data are not intended to replace existing benchmarks for biomechanical gait analysis, we believe they still fill a gap between those benchmarks and HAR datasets by providing richer neuromechanical data collected from wearable sensors using a unified protocol for several distinct locomotor activities. In this data report, we summarize our methods for instrumenting subjects, collecting data, and post-processing for artifact removal and gait segmentation. We also present a summary of the types of locomotor activities and transitions captured by our protocol, validate our results, and conclude with suggestions for how other researchers in the field may benefit from this dataset.

## Materials and Methods

### Instrumentation Setup

Ten healthy able-bodied subjects (seven male, three female; 25.5 ± 2 years; 174 ± 12 cm; 70 ± 14 kg) without any gait impairments were recruited and completed the following protocol between January and February 2017. Before walking, subjects were instrumented with wearable sensors to measure bilateral lower limb muscle activity and joint and limb kinematics. EMG signals were recorded using bipolar surface electrodes (DE2.1; Delsys, Boston, MA, USA) from the same seven muscles in each leg: tibialis anterior (TA), medial gastrocnemius (MG), soleus (SOL), vastus lateralis (VL), rectus femoris (RF), biceps femoris (BF), and semitendinosus (ST). These muscles were chosen because they are in part responsible for hip and knee flexion/extension and ankle plantarflexion/dorsiflexion, movements that are commonly assisted by wearable devices. They are also relatively easy to target when facing the subject from in front and behind. The muscle sites were prepared by removing excess hair, and the skin was cleaned by mildly scrubbing with an alcohol wipe. Sensors were attached to the skin with a double-sided adhesive. Electrode placement was guided by palpation according to the Surface ElectroMyoGraphy for the Non-Invasive Assessment of Muscles standards and verified by having subjects perform maximum voluntary contractions (MVC). Subjects performed three repetitions of ankle dorsiflexion/plantarflexion and knee flexion/extension for both legs. EMG signals were amplified by 1,000×, hardware band-pass filtered between 20 and 450 Hz (Bagnoli 16, Delsys), and sampled at 1 kHz.

**Figure 1:**
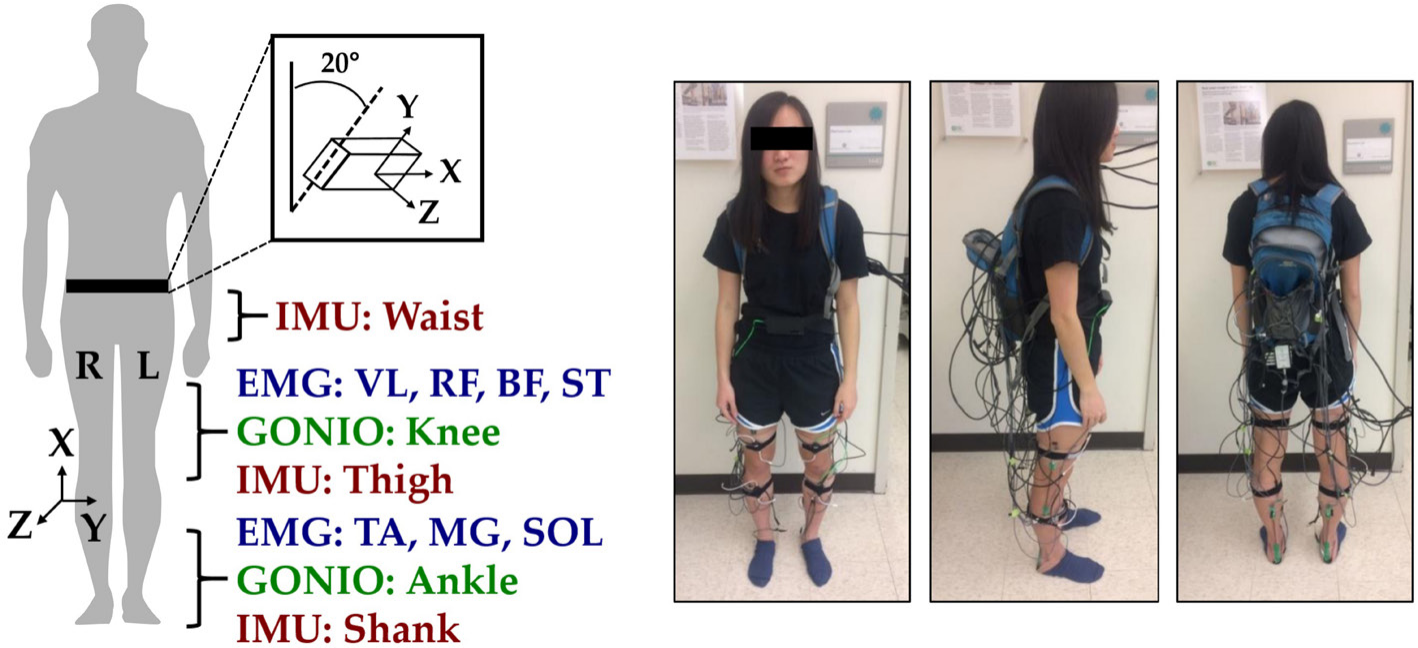
Instrumentation setup showing bilateral sensor placement. The orientations of the shank, thigh, and waist inertial measurement units (IMUs) are shown with coordinate axes. The subject provided written informed consent for the publication of this image.

Joint kinematic signals (sagittal plane only) were recorded using electrogoniometers (SG150; Biometrics Ltd., Newport, UK) placed on the knee and ankle and sampled at 500 Hz. At the beginning of trials, the goniometers were zeroed while the subject was in the upright standing position. 6-DOF (tri-axial accelerometer and gyroscope) inertial measurement units (IMUs) were placed bilaterally on the subjects’ thigh (below RF) and shank (adjacent to TA) and sampled at 500 Hz (MPU-9250; Invensense, San Jose, CA, USA). Goniometers and IMUs were secured to the subject using a combination of double-sided adhesive, elastic straps, and Coban self-adherent wrap. Another IMU was placed in a custom manufactured holster (tilted 20° from vertical) and worn around the waist with a belt. All signals were simultaneously recorded with a custom 16-bit data acquisition device that permitted multi-rate sampling. To facilitate integration with our custom data acquisition software, all wearable sensors were used in a tethered setup; as a drawback, fully instrumenting each leg took up to an hour. The full instrumentation setup with IMU orientations is shown for a representative subject in Figure 1.

### Data Collection Protocol

In an experimental session, each subject was barefoot and completed approximately 25 repetitions of a circuit consisting of sitting (S), standing (St), LW, ascending/descending a ramp with a 10° slope (RA/RD), and ascending/descending a four-step staircase (SA/SD) step-over-step. These activities were chosen because they encompass the different types of terrain likely encountered in community ambulation and were completed as a circuit in a 20 ft. × 30 ft. room for practicality and for increasing the number of repetitions. A platform (30″ tall) joined the staircase (7.75″ rise, 10″ run) and ramp (14 ft. long) to allow all possible transitions between these activities. Data from each circuit were divided into two segments and recorded as separate trials. Odd-numbered trials consisted of S → St → LW → SA → LW → RD → LW → St → S. Even-numbered trials consisted of S → St → LW → RA → LW → SD → LW → St → S. The total distance walked for each continuous segment was approximately 45 ft. Trials during which sensors needed to be repositioned or the tether became tangled were excluded. Subjects were instructed to freely transition between locomotor activities at their self-selected speed, and breaks were routinely administered to avoid fatigue. The experimenter labeled the true locomotor intent of the subject using a key fob. Data collection took up to 2 h.

### Post-processing

Heel contact and toe off gait events for each leg were reliably identified by finding peaks in the mean-subtracted and low-pass filtered (first-order Butterworth, 6 Hz) sagittal plane angular velocity (G_Y_) of the shank segment using a threshold-based method similar to Maqbool et al. (2016). Briefly, the largest peaks in angular velocity were first used to identify mid-swing events. Toe off events were identified by searching for peaks before each mid-swing event. Heel contact events were identified by searching for peaks after each preceding mid-swing event. Event switches were initially placed beneath the heel and first metatarsal of each foot, but they triggered many false negatives and positives in our setup perhaps due to mechanical wear and/or foot placement on the staircase. Therefore, they were only used for validating the IMU-based segmentation technique. Gait events corrupted by motion artifacts (i.e., pauses and trips) were excluded. EMG signals were high-pass filtered (sixth-order Butterworth) at 20 Hz, low-pass filtered (sixth-order Butterworth) at 350 Hz, and notch-filtered (sixth-order Butterworth, 6 Hz width) at 60, 180, and 300 Hz to attenuate motion artifact and ambient interference. Goniometer and IMU signals were low-pass filtered (sixth-order Butterworth) at 10 and 25 Hz, respectively. Joint velocities were indirectly computed by taking the central-difference numerical derivative of the joint position and added to the goniometer channels.

All signals were segmented into analysis windows beginning 300 ms before each identified heel contact or toe off gait event. Four additional 300 ms analysis windows near each identified gait event (delayed by 30, 60, 90, and 120 ms relative to each event) were used. For each window, we extracted features previously used in intent recognition for control of a powered knee-ankle prosthesis. Features for goniometer and IMU channels included the mean, SD, maximum, minimum, initial, and final values (Varol et al., 2010) (six features/channel). Features for EMG signals included the mean absolute value, waveform length, number of zero crossings and slope sign changes, and the coefficients of a sixth-order autoregressive model (Huang et al., 2005; Hargrove et al., 2008) (10 features/channel). There were a total of 23 sensors (14 EMG, 4 goniometer, 5 IMU), 52 channels (14 EMG, 8 goniometer, 30 IMU), and 368 features (140 EMG, 48 goniometer, 180 IMU).

## Results

The data are saved in CSV format in subject-specific folders and are available to download from Figshare at https://doi.org/10.6084/m9.figshare.5362627. Within each subject-specific folder, there is a metadata file, which catalogs the filenames, summary statistics (mean, SD, minimum, maximum) of each goniometer channel, and signal-to-noise ratios [ratio of maximum to baseline root-mean-square (RMS) voltage] of each EMG channel for each circuit. Subject-specific folders also include folders for the raw and processed data from individual circuits, a folder containing the processed EMG signals from all muscles during MVC trials, and a folder containing the features extracted from the five different 300 ms analysis windows (beginning 300, 270, 240, 210, and 180 ms before the gait events identified for each leg). Data from individual circuits also contain columns specifying the label of the true locomotor activity, the indices of heel contact and toe off gait events, and four-digit triggers denoting the outgoing and incoming locomotor activities and gait phases. The first row of each file is a header specifying the column order. The post-processed data from all trials are included for completeness although some trials include disturbances (e.g., pauses, trips, and missed transitions), which are noted in the metadata file. However, only gait events from disturbance-free segments of trials are reported and used for feature extraction. Feature data also contain columns specifying the corresponding leg-phase (1, right heel contact; 2, right toe off; 3, left heel contact; 4, left toe off) and the four-digit trigger. The first row of the feature data is a header specifying the column order. Ipsilateral refers to the side in which the gait event was detected (e.g., the right leg for right heel contact and right toe off events).

The overall composition of ENABL3S is shown in Table 1. For each subject, there were 530 ± 46 heel contact events and 536 ± 45 toe off events for each leg (mean ± SD) after excluding transitions to or from standing. Additional subject information and an explanation of nomenclature and numbering are also included on Figshare.

**Table 1:** Characteristics of ENcyclopedia of Able-bodied Bilateral Lower Limb Locomotor Signals.

	Transition to	Heel contact	Toe off	Total
Level walking (LW)	LW	4,523	4,637	9,160 (42.96%)
	RA	240	245	485 (2.27%)
	RD	240	246	486 (2.28%)
	SA	239	253	492 (2.31%)
	SD	248	243	491 (2.30%)
Ramp ascent (RA)	RA	1,408	1,416	2,824 (13.24%)
	LW	243	252	495 (2.32%)
Ramp descent (RD)	RD	1,757	1,762	3,519 (16.50%)
	LW	239	245	484 (2.27%)
Stair ascent (SA)	SA	489	472	961 (4.51%)
	LW	238	245	483 (2.27%)
Stair descent (SD)	SD	475	478	953 (4.47%)
	LW	248	242	490 (2.30%)
		10,587	10,736	21,323 (100%)

*The total number and proportion of gait events belonging to each type of locomotor activity are aggregated across all subjects*.

## Discussion

ENcyclopedia of Able-bodied Bilateral Lower Limb Locomotor Signals represents a benchmark of bilateral lower limb neuromechanical signals recorded from able-bodied individuals using wearable sensors during unassisted locomotion. The purpose of introducing this dataset is not to replace existing benchmarks for biomechanical gait analysis of steady-state locomotion but to provide a publicly available set of rich biomechanical data from wearable sensors, representing a compromise between traditional techniques and methods from HAR. ENABL3S includes data from several distinct walking-related activities (with transitions), which we expect to be helpful for understanding patterns in normal locomotion and developing novel control strategies for wearable lower limb assistive devices.

In order to assess the validity of this dataset, we chose to compare our recordings (averaged across legs and all subjects) to previously reported biomechanical measurements of level walking because these data are most accessible. Due to movement out of the sagittal plane and skin deformation/relative motion of the ankle goniometer, our measurements of ankle position were not considered biomechanically accurate signals. Nonetheless, these signals may still be useful for developing control strategies because many devices do not reproduce physiological motion and/or use embedded joint encoders to sense relative ankle position. However, our measurements of knee position were more accurate when compared to previously reported data recorded using optical motion capture (Winter, 1983; McClelland et al., 2011). The RMS error between ENABL3S and Winter (1983) was 5.9 and 9.5° for stance and swing phases, respectively. The *R*^2^ values were 0.73 and 0.94 for stance and swing phases, respectively. Our reported values for knee range of motion (ROM) during stance and swing phases (flexion at initial contact: 10.9 ± 5.6°; stance ROM: 6.8 ± 5.2 to 26.9 ± 5.7°; swing ROM: 3.4 ± 5.4 to 58.0 ± 6.5°) were also comparable to reported values (McClelland et al., 2011). Errors in position can be attributed to a combination of differences in walking speed, minor misalignment of the sensor with the axis of rotation, and skin deformation/relative motion. The knee position could also be estimated (perhaps more accurately) by subtracting the orientations of the shank and thigh IMU sensors. The patterns of EMG activation for ankle plantarflexor/dorsiflexor and knee flexor/extensor muscles were also qualitatively similar to those previously reported for unassisted overground walking at self-selected speed (Winter, 1983; Sylos-Labini et al., 2014). Knee position and EMG from TA, MG, BF, and VL aggregated across legs for all steady-state level walking steps for all subjects can be found in a supplementary document on Figshare. By confirming the accuracy of our measured kinematic and EMG signals, we also validate the IMU-based method for gait segmentation.

Although these data are not as high resolution as optical motion capture, they strike a balance between resolution of signals, breadth of activities represented, feasibility for online control schemes, and contribution to existing publicly available datasets for human locomotion. These data can be used for developing novel control strategies such as intent recognition (i.e., predicting future states based on signals detected before movement completion) and more specifically investigating sensor fusion techniques and machine learning approaches for feature extraction and classification (e.g., deep learning). These data can also be interpreted as a simulation of able-bodied individuals walking with a completely massless and transparent (i.e., perfectly backdrivable) device and can be used to derive a device-agnostic upper bound on control strategies such as intent recognition. The raw data reported here may also be useful for comparing the performance of alternative control systems, assessing inter-subject variability, and comparing user-based biomechanical signals collected from an impaired population or a population walking with an assistive device (e.g., knee orthosis and ankle-foot prosthesis) to unassisted normal locomotion.

## Ethics Statement

This study was carried out in accordance with the recommendations of the Northwestern University Institutional Review Board with written informed consent from all the subjects. All subjects gave written informed consent in accordance with the Declaration of Helsinki. The protocol was approved by the Northwestern University Institutional Review Board.

## Author Contributions

BH helped in conceiving the study concept, collecting, analyzing, and interpreting the data, and drafting the manuscript. LH and ER helped in conceiving the study concept and interpreting the data, critically revising the manuscript for important intellectual content, obtaining funding, and supervising the study. All the authors read and approved the final manuscript.

## Conflict of Interest Statement

The authors declare that the research was conducted in the absence of any commercial or financial relationships that could be construed as a potential conflict of interest.
