# Corrigendum: Benchmark Datasets for Bilateral Lower-Limb Neuromechanical Signals from Wearable Sensors during Unassisted Locomotion in Able-Bodied Individuals

**DOI:** 10.3389/frobt.2018.00127

**Published:** 2018-11-20

**Authors:** Blair Hu, Elliott Rouse, Levi Hargrove

**Affiliations:** ^1^Center for Bionic Medicine, Shirley Ryan AbilityLab, Chicago, IL, United States; ^2^Department of Biomedical Engineering, Northwestern University, Evanston, IL, United States; ^3^Department of Mechanical Engineering, University of Michigan, Ann Arbor, MI, United States; ^4^Department of Physical Medicine and Rehabilitation, Northwestern University, Chicago, IL, United States

**Keywords:** gait, locomotion, biomechanics, electromyography, benchmark

In the original article, there were two errors. In the text, the abbreviation for semitendinosus was omitted. In the text, the URL to the data repository available on Figshare was also incorrect.

Corrections have been made to Materials and Methods, Sub-section Instrumentation Setup, Paragraph one and Results, Paragraph one.

EMG signals were recorded using bipolar surface electrodes (DE2.1; Delsys, Boston, MA, USA) from the same seven muscles in each leg: tibialis anterior (TA), medial gastrocnemius (MG), soleus (SOL), vastus lateralis (VL), rectus femoris (RF), biceps femoris (BF), and semitendinosus (ST).

The data are saved in CSV format in subject-specific folders and are available to download from Figshare at https://doi.org/10.6084/m9.figshare.5362627.

The authors apologize for these errors and state that they do not change the scientific conclusions of the article in any way. The original article has been updated.

## Conflict of interest statement

The authors declare that the research was conducted in the absence of any commercial or financial relationships that could be construed as a potential conflict of interest.

